# Global challenges in aging: insights from comparative biology and one health

**DOI:** 10.3389/ftox.2024.1381178

**Published:** 2024-05-30

**Authors:** Mary Ann Ottinger, Jacquelyn K. Grace, Terri J. Maness

**Affiliations:** ^1^ Department of Biology and Biochemistry, University of Houston, Houston, TX, United States; ^2^ Department of Ecology and Conservation Biology, Texas A&M University, College Station, TX, United States; ^3^ School of Biological Sciences, Louisiana Tech University, Ruston, LA, United States

**Keywords:** one health, toxic coin, exposome, wildlife health, ecosystem resilience, sustainability, pollution, human health

## Abstract

The well-being of wildlife populations, ecosystem health, and human health are interlinked, and preserving wildlife is crucial for sustaining healthy ecosystems. Wildlife numbers, and in particular avian populations, have steeply declined over the past century, associated with anthropogenic factors originating from industry, urbanization, changing land use, habitat loss, pollution, emerging diseases, and climate change. All these factors combine to exert increasing stress and impair health for both humans and wildlife, with diminished metabolic, immune, and reproductive function, deteriorating overall health, and reduced longevity. The “toxic aging coin” suggests that these stressors may have dual impacts on aging–they can accelerate the aging process, and older individuals may struggle to cope with pollutants compared to younger ones. These responses are reflected in the health and productivity of individuals, and at a larger scale, the health and ability of populations to withstand disturbances. To understand the potential risk to health over the lifespan, it is important to articulate some of these global challenges and consider both their impacts on aging populations and on the aging process. In this review, we use the toxic aging coin and One Health conceptual frameworks to examine the interconnected health of humans, wildlife, and ecosystems. This exploration aims to develop proactive approaches for optimizing wildlife and human health.

## 1 Introduction

The challenges humanity faces today result from complex, simultaneous events often driven by human activities, along with their side effects and unintended consequences. The advances made by humanity in technology and industrialization have improved many of the characteristics of human life and lifespan. However, coincident with these advances, are environmental and lifestyle changes that affect individuals’ health and life spans. While these challenges primarily concern humans, the conditions affecting humans and their communities also have repercussions for wildlife and ecosystems. The well-being of wildlife populations, ecosystem health, and human health are interlinked, and preserving wildlife is crucial for sustaining healthy ecosystems. This interrelationship and interdependence of human-wildlife-ecosystem is captured in the One Health concept, which provides a broad conceptual framework that brings together these dynamic factors, including exposure to environmental contaminants. The “toxic aging coin” provides a perspective to consider these challenges within the context of an aging population. On one side we find that older individuals may experience different effects from toxicants and other environmental changes, and on the other side we see that toxicants can accelerate the aging process (Wise, 2019). While emphasis has been placed by other authors on investigating the toxic aging coin in laboratory toxicology research (Wise, 2019), this perspective is just as important outside of the laboratory, including public health, ecology, and other “field” research. This review will focus on select global challenges relevant to the toxic aging coin perspective, setting the context for how all species, including aging individuals, must cope.

Various consortia, think tanks, and international organizations have compiled lists of global challenges. These lists differ in their level of specificity, ranging from highly detailed to broad overviews. First, the Millennium Project listed 15 Global Challenges that include the following: sustainable development and climate change, clean water, population and resources, democratization, global foresight and decision making, global convergence of IT, rich-poor gap, health issues, education and learning, peace and conflict, status of women transnational organized crime, energy, science and technology, and global ethics (Glenn et al., 2017). This list is thorough with regard to physical climate, population, and resource concerns as well as social issues. A second list, which includes: climate change, ecological collapse, weapons of mass destruction, emerging and unknown risks, and natural catastrophes, compiled by The Global Challenges Foundation (Global Challenges Foundation, 2024) focuses on the complexities of climate change, and large-scale environmental degradation from the aspects of drivers, specifically population growth, poverty, and political and social factors in key regions.

The list prepared by the United Nations (UN) prioritizes activities in peace and security, human rights, humanitarian aid, sustainable development and climate action, international law, and global issues (United Nations, 2024a). The UN identified 17 sustainable development goals: no poverty, zero hunger, quality education, good health and well-being, gender equality, sustainable cities and communities, climate action, and partnerships (United Nations, 2024b). UN global issues (United Nations Global Issues, 2024) encompass 23 goals, some of which include: Africa, aging, AIDS, atomic energy, big data for sustainable development, children, climate change, democracy, disarmament, ending poverty, food, migration, human rights, migration, peace and security, and water. Highlighting just a few of the many of the UN’s sustainable development, climate action, and global issues goals demonstrates the cross-cutting nature of these challenges facing humanity. Furthermore, each of these goals and issue areas are multi-layered, with many contributing factors and modulators. In the case of sustainable development, the data tracker from the Our World, (2024) provides comprehensive information about a range of topics (demographic change, health, food and agriculture, energy and environment, innovation and technological change, poverty and economic development, living conditions, community and wellbeing, human rights and democracy, violence and war, education and knowledge), which are constantly updated. Further, the Toxic Release Inventory (TRI) program provides an outstanding resource for tracking the release of pollutants and the characteristics of these chemicals (United States Environmental Protection Agency, 2024). It is clear that these issues are global and the nature of these topics illustrates the complexity of the challenges facing humanity, including longevity and healthy aging, which is applicable to both human and wildlife populations. We focus on a few of these drivers that are pertinent to the toxic aging coin perspective, and how the One Health concept may provide a structured intervention framework.

## 2 Global challenges affecting healthy aging

First, we consider the history and context in which we find ourselves. As humans, we have acquired greater capabilities through advanced technology and industrialization. Consequently, the threats and challenges facing humanity have become increasingly global. The Covid-19 pandemic illustrates the globalization of our national and community connectivity, both for the many benefits as well as the commensurate risks. Identifying and characterizing the factors that catalyze the emergence and influence the power of global threats to humanity, wildlife, and ecosystems is a crucial need. Understanding the critical needs that overwhelm the risks and transcend national borders is equally imperative. We examine these compelling areas of change in human society and consider the impacts on human-wildlife-ecosystem health with specific attention to both aging populations and the effects of these changes on the aging process, which has become an important focus for human health (Nwanaju-Enwerem et al., 2021; Malecki et al., 2022). Our discussion reflects human needs and our response to industrialization and technology throughout the world, with coincidental effects on planetary One Health and individual Exposomes. We provide some data to show change over time and to illustrate interconnections of relevant factors. We have selected several large challenges: global linkages—transport of people and goods, shifting global demographics, and resources and dynamic factors contributing to deteriorating environmental quality. All these challenges affect healthy aging in populations of humans and wildlife.

### 2.1 Global linkages—transport of people and goods

**FIGURE 1:**
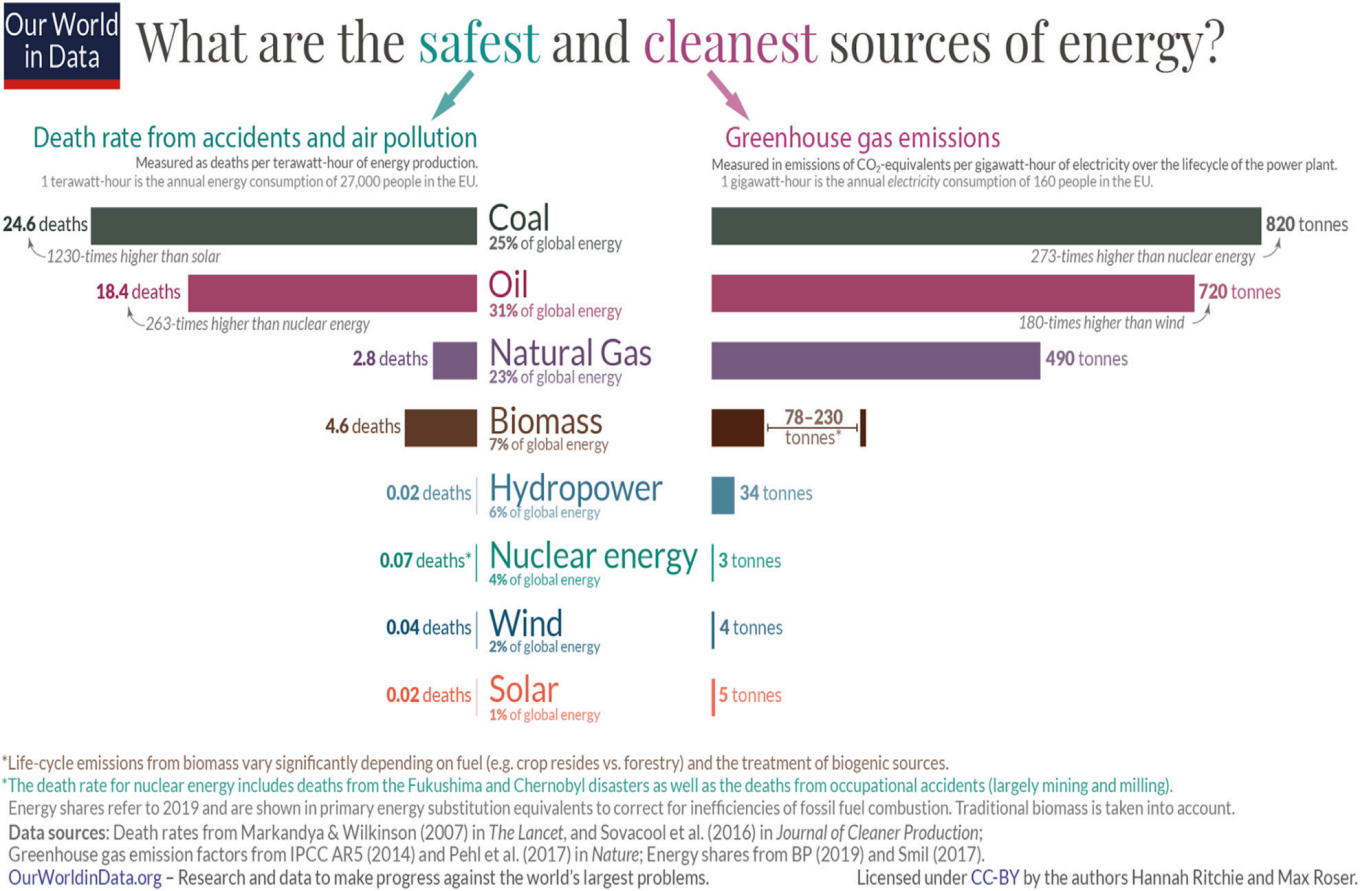
Comparison of the safety and environmental impacts, as measured by greenhouse gas emissions for various sources of energy (from Our World in Data, available through CC-BY by the authors, Ritchie and Rosner).

Transportation has undergone tremendous change over the past century, shifting from predominantly animal, coal, and steam powered mechanisms to fossil fuel powered cars, trucks, trains, ships, and airplanes for movement of people and goods (Ortiz-Ospina and Beltekin, 2018). These energy sources differ in the potential for environmental impacts due to different pollutants being released during production and use. Recently, “clean energy” has received growing attention, given the issues with pollution (Figure 1), increasing energy consumption, and geopolitical unrest.

**FIGURE 2:**
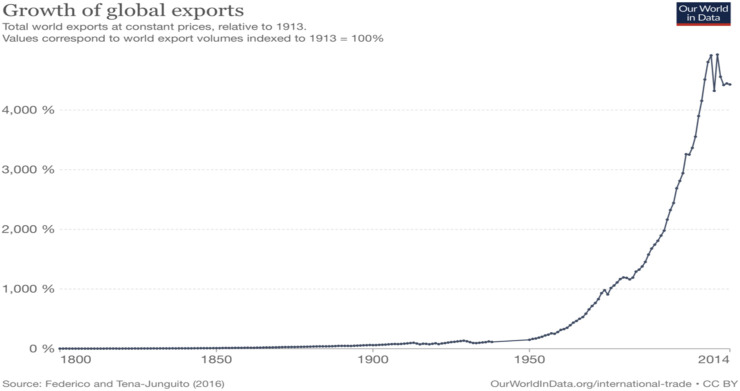
Visualization of the growth in exports globally shows a sharp increase in the past 70 years (from OurWorldinData.org/internationaltrade; https://ourworldindata.org/trade-and-globalization#).

Beyond the transport of people, global trade has grown exponentially (Figure 2; Ortiz-Ospina and Beltekin, 2018). Further information is available from an interactive data visualization program Our World in Data showing cargo shipping across oceans, with carbon footprint, containers, materials transported (Ortiz-Ospina and Beltekin, 2018). Together these data show the global nature of trade and movement of people. In addition, they illustrate the sharp increase in the interconnectedness of nations world-wide. Accordingly, solutions will rely on internationally coordinated programs and interventions to reduce the movement of invasive species, disease, and other problematic globally transported entities.

A critical threat associated with the movement of products is the demand for illegal goods and services. Beyond the appalling and complex issues associated with human trafficking, poaching and trafficking wildlife has decimated wildlife (Gore et al., 2020; 2023). The illegal trafficking of live animals, and their products can spread disease and toxicants to humans and wildlife (Machalaba et al., 2019; Borsky et al., 2020; Machalaba et al., 2020; Shivaprakash et al., 2021). Ecosystems have borne the brunt of this emerging tragedy, becoming increasingly disrupted due to illegal overharvesting of wildlife with concomitant alterations in food webs and ecosystem function. Trafficking is one of the underlying causes for measurable loss in many species, with some of the most egregious being for populations of rhinoceros, vultures, pangolins, and bats (Wingard et al., 2020). These losses occur in both species targeted by poachers and those not targeted. For example, one of the greatest threats to vulture populations is poisoning by humans for consumption or cultural/religious practices, but also to decrease the visibility of poached rhinoceroses’ carcasses. Poachers will poison the remaining carcass of a rhino or other poached animal to remove these vultures that serve as sentinels of poaching activities within federal parks (Gore et al., 2020). Additional negative consequences to humans from these activities include poisoning from the residue on vultures that may sicken or kill humans or other animals ingesting the carcasses or using the products (feathers, head, feet, and others); exposure to zoonotic pathogens on the feathers or parts of the carcass; diminished ‘clean up’ of carcasses thereby increasing the risk from anthrax and other zoonotic diseases. This illustrates some of the many adverse health outcomes from poaching and trafficking on ecosystems, wildlife populations, and human health and communities. Trafficking can also lead to the spread of invasive species, which poses a significant risk to indigenous wildlife by supplanting their niche in the ecosystem and upsetting ecological balance, food networks, and ecosystem stability (Gore et al., 2020). Some of invasive species transported through trade and containers on ships (USDA, 2024)) also carry diseases. Thus, the movement of people and goods poses several challenges to human and wildlife populations including the increased spread of disease, increased rates of pollution associated with fossil fuel as a transportation energy source, and the introduction of non-native species and removal of native species from ecosystems (Borsky et al., 2020; Machalaba et al., 2020; Shivaprakash et al., 2021). The overarching question is how these global challenges, with their anthropogenic impacts affect human and wildlife populations and importantly if aging individuals are more adversely affected than younger populations. We consider some of these questions in more detail below.

### 2.2 Shifting global demographics

**FIGURE 3:**
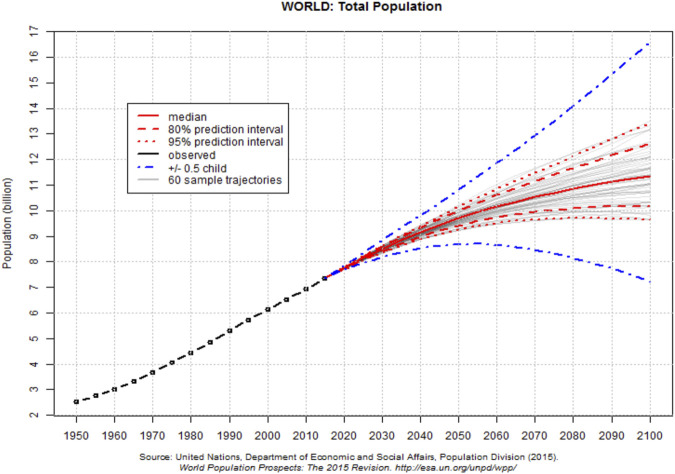
Estimated probabilistic projections for world population (from: United Nations, 2024; https://esa.un.org/unpd/wpp/Graphs/DemographicProfiles/; https://esa.un.org/unpd/wpp/Graphs/Probabilistic/POP/TOT/).

As the global human population grows and expands, land use, economic stability, medical services, and multiple community and regional systems become increasingly stressed and overwhelmed (Dodson et al., 2020; Degroot et al., 2021). These impacts are also felt world-wide, with more developed countries often attempting to provide support through national and international agencies and by multiple non-profit, religious, and social organizations. The UN Department of Economic and Social Affairs, Population Division has provided historical data and probabilistic projections that show increasing trends in population from 1950 to 2,100 (Figure 3; United Nations Department of Economic and Social Affairs, Population Division, 2024). Key factors necessary for human health are also impacted by population growth, with increasing density equating to increasing disease, pollution, and other potential risks. These factors influence the ability of an individual to maintain health and resilience throughout their lifespan.

**FIGURE 4:**
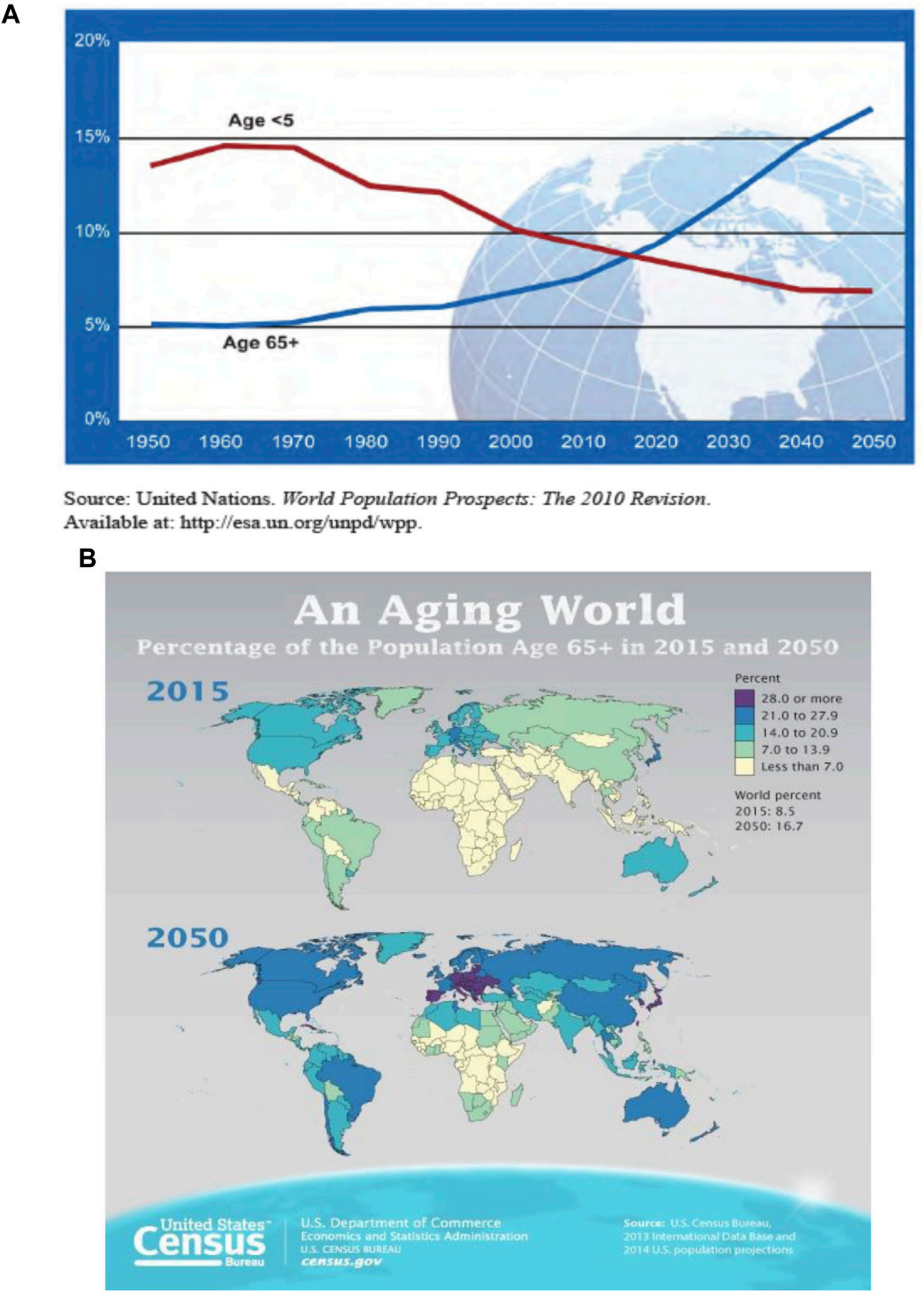
**(A)** Number of children and aging adults as a percentage of the global populations (from United Nations World Population Prospects (2024); **(B)** Global increase in aging individuals (from US Census Bureau, 2016).

**FIGURE 5:**
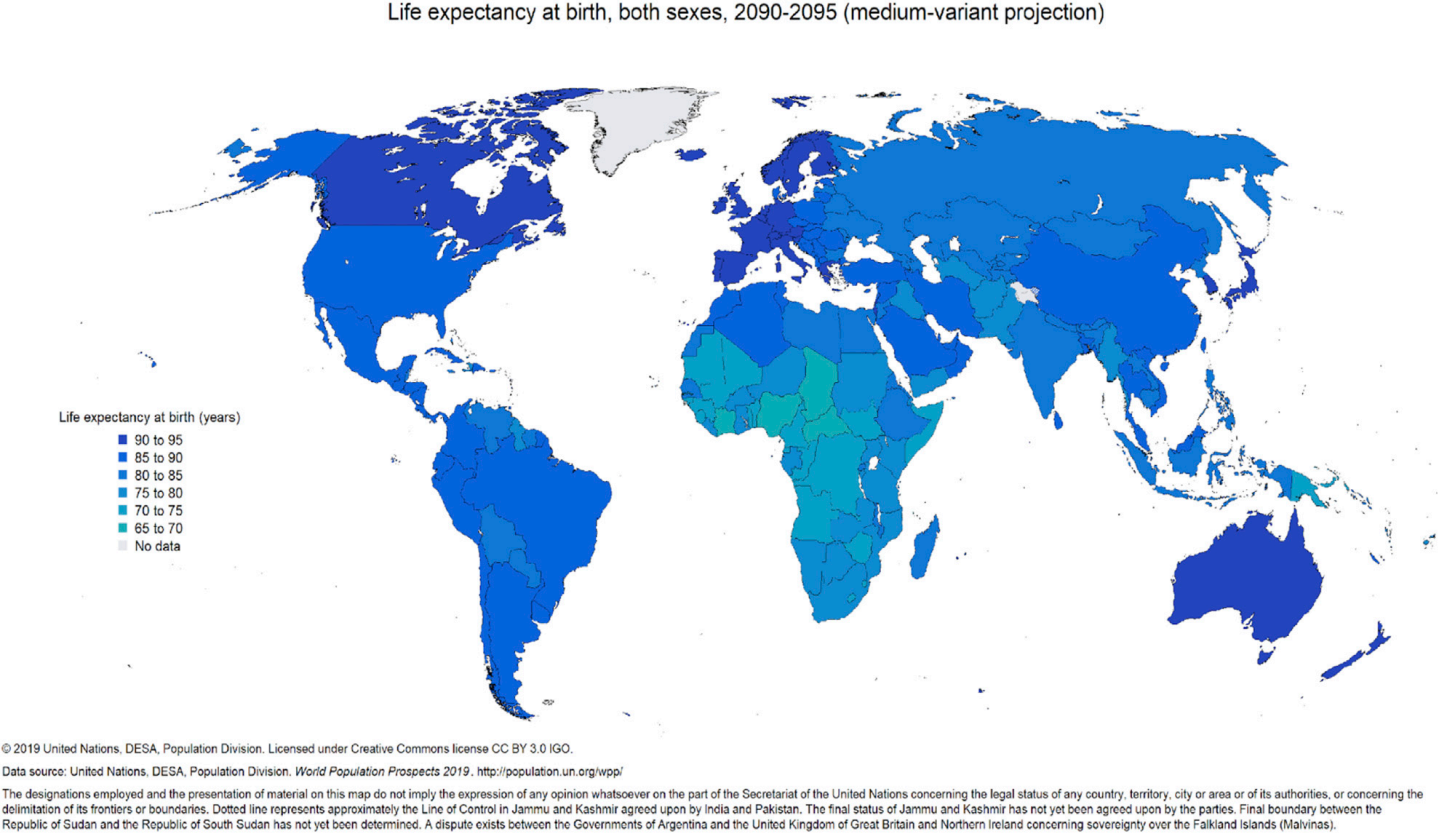
Global distribution of predicted life expectancy for both genders at birth 2090-2095 (from UN Population Division; https://population.un.org/wpp/Maps/6.1_Life%20Expectancy%20-%20Both%20Sexes/e0Total-LowRes-2090.png).

A primary contributor to increasing global population growth is the decline in death rates and associated increase in expected lifespan (Bongaarts, 2009). Our progress in medical sciences has had the fortunate outcome of longer lifespan in many areas of the world (Suzman and Beard, 2011; United Nations, 2020b). While there is considerable diversity in longevity and aging characteristics among communities, regions, countries, and continents, there is a general global trend toward an increased number of elderly individuals (Figure 4A). Further, the increase in the aging population is not evenly distributed across the globe. Instead, projections indicate a higher concentration of aging individuals in Europe, widespread increases in North America, Australia, and Asia, with scattered concentrations of increased lifespan in regions of South America and Africa (Figure 4B), a trend that is predicted to continue into the late 21st century (Figure 5). Global migration patterns can contribute to the relative age distribution in the countries of origin and destination, with downstream effects on the overall health of aging populations. Migrants tend to be young, working age individuals, and help to offset the imbalance of younger to older people in more developed countries (Münz, 2013). Between 2000 and 2020, Europe, North Africa and Western Asia collectively received the largest number of international migrants, followed by North America, with the United States having a large share of migrants (United Nations Department of Economic and Social Affairs, 2020a). However, these trends may shift as new economic opportunities open in migrant home countries or other nations (e.g., China and Korea; Münz, 2013). Regardless of these local shifts in demographics, however, the global trend remains toward an increasingly aged population. This shift in demographics will have, and is already having, economic and societal impacts associated with medical care, daily sustenance, workforce capabilities, and overall community structure and function (for more information, see United States Census Bureau, 2016; United Nations, 2020b). Medical care is an especially critical concern for the elderly, who are at greater risk of physical and cognitive decline and disability (De Luca d’Alessandro et al., 2011. Globally, aging is projected to surpass population growth as the primary demographic cause of health expenditure growth by 2050, and already is the main demographic driver of health expenditures in more developed countries (Mayhew, 2000).

### 2.3 Resources and dynamic factors—climate and critical resources

**FIGURE 6:**
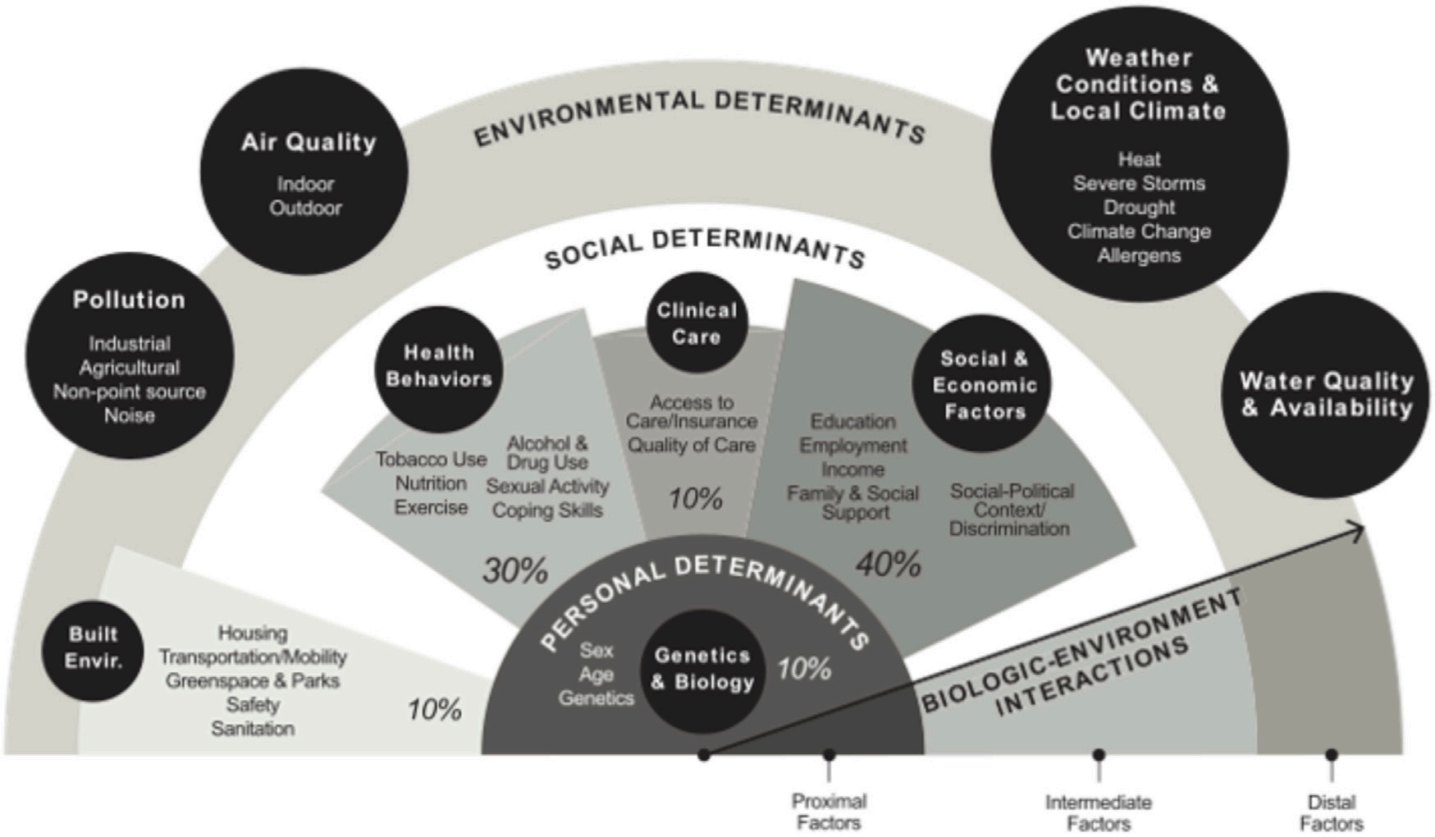
Determinants of health for individuals in the 21st century (percentages illustrate relative impacts, also influenced by local climate, water and air quality, and pollution (from Ottinger and Geiselman, 2023).

Numerous factors influence both the lifespan and, importantly, the healthspan of an individual. The environmental modulators for personal health of an individual can be diagrammed according to life stages and probable exposures, which act upon the backdrop of an individual’s genetic make-up and physiology (Dagnino, 2019). Resource availability is regionally and community specific, also being dictated by many dynamic factors, including political, social, and cultural, geolocation, and environmental (e.g., pollution, industry, availability of goods and services, education). A vast array of resources is needed to maintain healthy humans, wildlife, and ecosystems, including access to clean water, and high-quality food, habitat, refuge/shelter, and healthcare for humans. This involves optimizing environmental and social determinants to promote optimal health and overall welfare (Figure 6). Many of these same factors affect wildlife and ecosystem health and as such, are closely tied to human health. Over the past century, humans, wildlife and ecosystems have been constantly barraged by dynamic changes to these resources associated with land use, pollutants, human communities, climate change, and other factors (Degroot et al., 2021; Benham and Bowie, 2023). For example, urbanization is a primary driver of biodiversity loss, globally, due to loss of habitat, food, and other resources (especially for specialist species), followed by biotic and seasonal homogenization in urban environments (McKinney, 2006; Leveau et al., 2021; McCloy et al., 2022; 2024). Urbanization also poses challenges for humans, especially when urban growth is rapid and/or poorly managed. Poor urban development can lead to inadequate clean water, unsafe housing, poor sanitation practices, increased risk of disease due to crowded conditions, and elevated pollution levels (Aliyu and Amadu, 2017). Climate change is an additional dynamic factor with huge and varied effects on human, wildlife, and ecosystem resources. The climate is currently warming at unprecedented speed, resulting in a plethora of climatic changes, including more frequent heat waves and droughts, increased precipitation over land and more frequent intense precipitation events, sea level rise, shifts in climate zones, and changes to storm tracks (Masson-Delmotte et al., 2021). The effects of these changes are already being felt globally, with the strongest effects in some of the world’s poorest countries (Mendelsohn et al., 2006). For both humans and wildlife, climate change is impacting food and water supplies, access to safe housing/habitat, disease transmission and other health concerns. Developing effective and coordinated frameworks is critical to effective programming to address global issues. To be successful, there must be coordinated programmatic approaches at regional, national, and international levels.

### 2.4 Global challenges from a toxic aging coin perspective

This brief overview of selected global challenges is intended to lay out the outcomes to humans, wildlife, and ecosystems that accompany industrial and technical advances. Each challenge is complex, with many layers of influencing factors and critical components that contribute to its emergence and effects. Each challenge is pertinent to both One Health and the Exposome frameworks because the threats and issues raised have deleterious effects on all aspects fundamental to each conceptual framework (Ottinger and Geiselman, 2023). Each challenge may also have age-dependent impacts on humans and wildlife, while also accelerating aging and many of its associated health issues. As the global human population shifts further and further toward older individuals, there is an increasing risk for adverse effects from the myriad of environmental challenges facing global human populations. Similarly, impacts of these challenges on wildlife may also be age-dependent and/or affect rates of biological senescence with long-term implications for population structure and stability. However, there is an enormous data gap in understanding aging in wild populations and in addition the potential effects of anthropogenic influences/insults on wildlife and ecosystem resilience (Benhan and Bowie, 2023). Within the One Health framework, which recognizes the interconnected nature of our human-wildlife ecosystems, we can develop further understanding of these challenges and their implications for long-term health and resilience of wildlife and ecosystems.

## 3 Lessons learned from the comparative biology of aging

To illustrate how the toxic aging coin perspective relates to wildlife, we present two aspects in which research in the comparative biology of aging provides critical information that is relevant to human biomedicine and to wildlife sustainability and conservation. The first dimension is that the knowledge that we gain from studies of comparative biology of aging is valuable for biomedical insights (Comizzoli and Ottinger, 2021). In addition to the laboratory animals generally used in research on aging, there are valuable insights to be gained from studying comparative models (Ottinger and Geiselman, 2023). These models include both invertebrate and vertebrate species that have unique characteristics, such as regeneration of organs, negligible aging, resistance to oxidative damage, variable life histories, susceptibility to specific cancers or other diseases, and cell systems (de Sousa et al., 2023; for reviews across models, see Ram and Conn, 2018). Geroscience, which encompasses research and applications for human biomedicine and aging now uses these many novel animal models to ask specific questions pertinent to biomedical insights and applications. Understanding the effects of environmental stressors, including specific toxicants on these animal models of aging will reveal aspects of the toxic aging coin. Comparisons across vertebrate and even invertebrate species reveal many conserved mechanisms that also occur in humans; thereby providing many insights into human aging processes by studies on other long-lived species (Ellison and Ottinger, 2014; Ottinger, 2018). Age-related deterioration in metabolic and immune function is often accompanied with declining cognitive function. A fundamental characteristic of aging in many individuals is the development of metabolic syndrome, in which a slowing metabolism is accompanied by increasing insensitivity in glucose modulation and impaired energy utilization. Metabolic and neurodegenerative diseases often have altered inflammatory responses, with deleterious effects on healthy aging and quality of life (Spann and Ottinger, 2018).

The second aspect is the importance of understanding wildlife species, including their biology, position in the food chain and ecosystem, and resource needs. All become critical in sustaining and preserving wildlife populations and their health, which then affects the resilience of ecosystems. The loss of biodiversity associated with anthropomorphic effects from human activities has accelerated over the past century, with stunning losses of many species. This clearly illustrates the deleterious effects of the byproducts and effects industrialization and technological advances on the environment and more specifically global, regional, and local ecosystems. Because wildlife species have a range of life histories, it is important to obtain information about their life history, resource needs, and adaptive strategies that enable them to survive and reproduce successfully. These data will contribute to critical programs designed to mitigate environmental factors that underlie the toxic aging coin and are likely to limit the survival as well as productivity of many vertebrate and invertebrate species. Finally, information on the key ingredients necessary to encourage health and resilience in wildlife will also contribute to the improvement of global ecosystems, including human communities. There are implications for diminished reproduction, increased disease, and decreased lifespan in wildlife from anthropogenic associated impacts (Ottinger and Geiselman, 2023). The toxic aging coin is highly pertinent for wildlife, given the effects of these stressors on population health and productivity, and the overall future status of wildlife populations.

### 3.1 Effects of environmental stressors on birds

**FIGURE 7:**
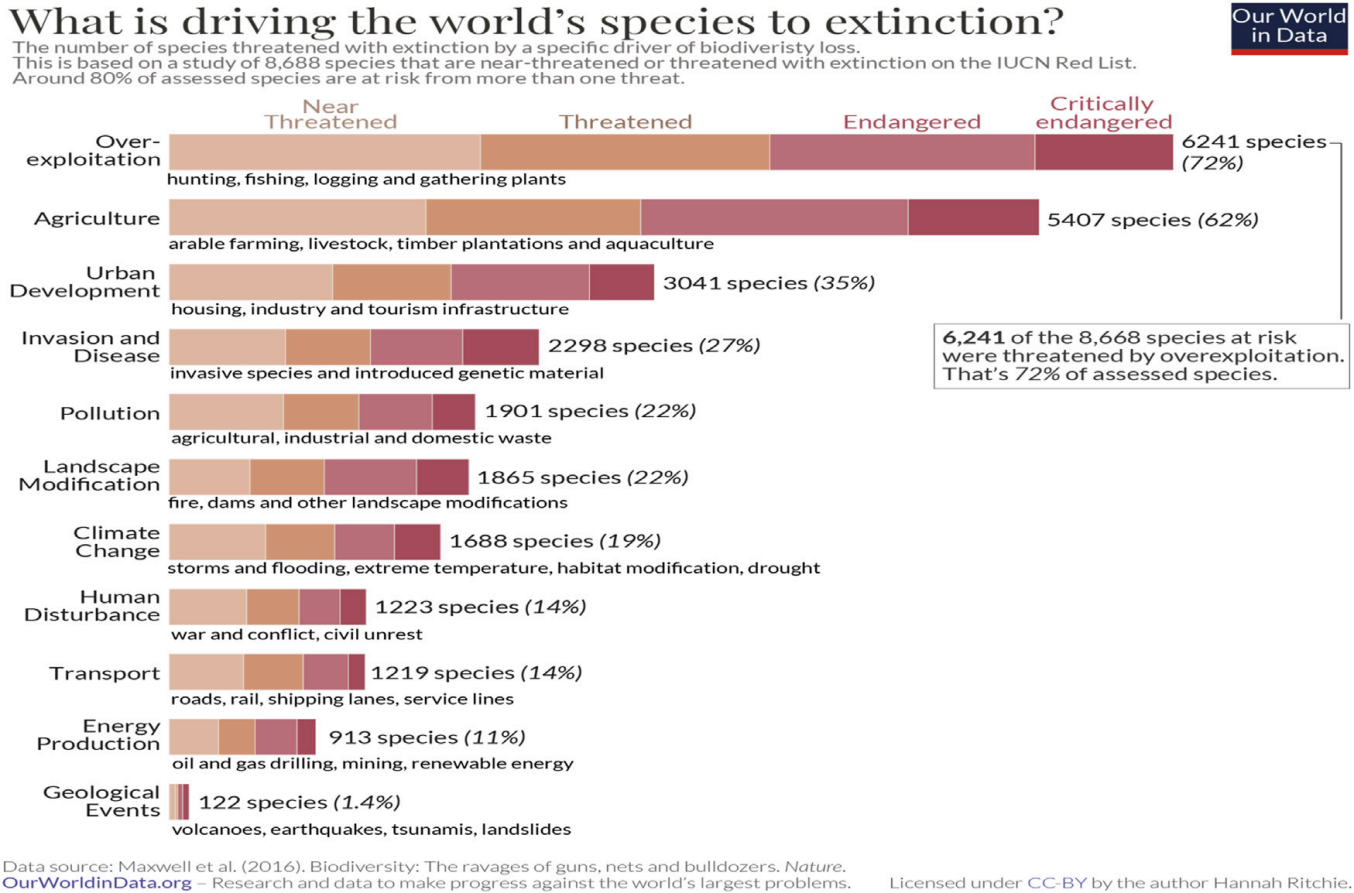
Threats to wildlife have varied sources, including agriculture, environmental chemicals, habitat loss, pollutants, and climate change (Ritchie and Roser, 2021).

Wildlife populations have steeply declined over the past century, associated with anthropogenic factors originating from industry, urbanization, changing land use, habitat loss, pollution, emerging diseases, and climate change (Figure 7). All combine to exert increasing stress and impair health, including diminished metabolic, immune, and reproductive function, deteriorating overall health, and reduced longevity. Birds have shown an exceedingly steep demise in their population numbers and an increase in the number of species that have become threatened and endangered (U. S. North American Bird Conservation Initiative, 2024). The health of avian populations, ecosystem health, and human health are inexorably linked, and wildlife conservation is fundamental to sustaining healthy ecosystems. Birds are frequently used as bioindicator and sentinel species because they are well studied, easy to observe in nature, conspicuous to the public and perform a diverse array of ecological functions.

## 4 Effects of global challenges on humans and birds: a toxic coin perspective

Recent changes to the global transport of people and goods, investment in fossil fuels as an energy source, the rapid increase in the global human population, and thus pose a number of challenges to humans and ecosystems, including increases in the spread of disease (both zoonotic and non-zoonotic) and widespread increases in environmental toxicants. These challenges can affect aging populations in ways that are different from the effects on younger populations, while simultaneously accelerating the aging process. For example, the COVID-19 pandemic caused by the SARS-CoV-2 coronavirus rapidly became a global crisis (Umakanthan et al., 2020). Older individuals (>65 years of age) are more vulnerable to COVID-19 and experience higher mortality rates, although the rate of increased mortality varies by country (Yuki et al., 2020; Biswas et al., 2021; Sasson, 2021). This appears to be due to declines in natural immunity, increased vulnerability to adverse drug reactions, decreased organ function, and increases in comorbidity factors with age, among other possible factors (Biswas et al., 2021). Younger individuals who have been infected with COVID-19 may also experience long-term effects including fatigue, hair loss, attention disorders, and dyspnea (Lopez-Leon et al., 2021), which are also associated with aging (e.g., Lufi et al., 2015; Buarque et al., 2022) and possibly an indicator of accelerated biological aging. Similarly, aged non-human vertebrates can also exhibit elevated vulnerability to disease due to immunosenescence (Lavoie et al., 2007) and/or increased exposure to vectors of disease (Bennett and Fallis, 1960). For example, risk of avian malaria and other haemosporidian infection generally increases with age for birds, as has been shown through cross-sectional studies in a variety of species in west Texas (Martinez et al., 2023) and the Missouri Ozarks (Ellis et al., 2014), white-banded tanagers (*Neothraupis fasciata*) in Brazil (Fecchio et al., 2015), blue tits (*Cyanistes caeruleus*) in Sweden (Podmokla et al., 2014) and the United Kingdom (Wood et al., 2007), and in at least one longitudinal study of house martins (*Delichon urbica*) in Spain (Marzal et al., 2016). On the other side of the toxic coin, infection with avian malaria appears to accelerate aging in at least one bird species, the great reed warbler (*Acrocephalus arundinaceus*), as evidenced by accelerated telomere shortening, decreased reproductive success, and decreased longevity (Asghar et al., 2015).

Fossil fuel combustion is the primary form of energy generation for modern transport and is a major contributor to air pollution (e.g., particulate matter, nitrogen oxides, sulfur dioxide, ozone, mercury), which can have disproportionate effects on both the young and elderly (Perera, 2018; Sykes, 2021). Older adults have higher rates of chronic heart and lung disease, which can be aggravated by air pollution, leading to increased hospitalization, emergency room visits, medication usage, and death. Chronic exposure to air pollution has also been associated with impaired mental abilities and dementia in older adults (reviewed in Sykes, 2021). Particulate matter and nitrogen oxides may also increase the spread and lethality of COVID-19, interacting to amplify the adverse effects of each of these challenges on aging populations (Copat et al., 2020). On the flip side of the toxic aging coin, air pollution can also accelerate cardiovascular aging, through increases in cardiovascular disease, cardiovascular oxidative stress, and inflammation (reviewed in Kuntic et al., 2023). Traffic-related air pollution is also associated with premature skin aging (reviewed in Schikowski and Huls, 2020), autophagy aging disorders (Numan et al., 2015), and generally accelerated aging and increased age-related diseases through associations with elevated oxidative stress and inflammation (Hahad et al., 2021). Similar effects have also been observed in wildlife, although longitudinal or cross-sectional studies evaluating age-dependent effects are limited. In birds, air pollution is associated with increased oxidative stress, decreased immune function, increased susceptibility to disease, changes in respiratory morphology and function and advanced telomere shortening (Barton et al., 2023), all of which are associated with premature aging.

Plastics are an additional contaminant associated with rapid human population growth, industrialization, and urbanization that may threaten human and wildlife health. Plastics pose a physical and ecotoxicological concern for humans (Prata et al., 2020) and wildlife, including birds (Grace et al., 2022). Plastics can carry chemicals that were used in the production process, as well as those adsorbed from the environment (Grace et al., 2022). These chemicals are associated with several indicators of accelerated aging in model species, including increased oxidative stress (Tan et al., 2015), DNA damage (Shin et al., 2019), inflammation (Prata et al., 2020), and pulmonary toxicity (Danso et al., 2022). Nanoplastics also appear able to cross the blood-brain barrier in mice, contributing to the onset of neurological disease including Parkinson’s disease and related dementias (Liu et al., 2023). Given the highly conserved nature of these physiological processes, it is likely that these effects would occur in humans and vertebrate wildlife. In birds, microplastics are associated with damage to the gastrointestinal tract (Charlton-Howard et al., 2023) which may accelerate organ aging. Microplastics may also increase the spread of disease (Beans, 2023) while decreasing disease resistance by inhibiting the innate and acquired immune responses (Yan et al., 2024). Given the high rate of comorbidities, including lung disease, and immunosenescence in aging populations, it is likely that these adverse effects would be felt more strongly in aged individuals, or would amplify existing health issues. Further research in geriatric populations is thus highly warranted.

## 5 One Health and the exposome frameworks to combat the effects of environmental stressors on humans and wildlife

**FIGURE 8:**
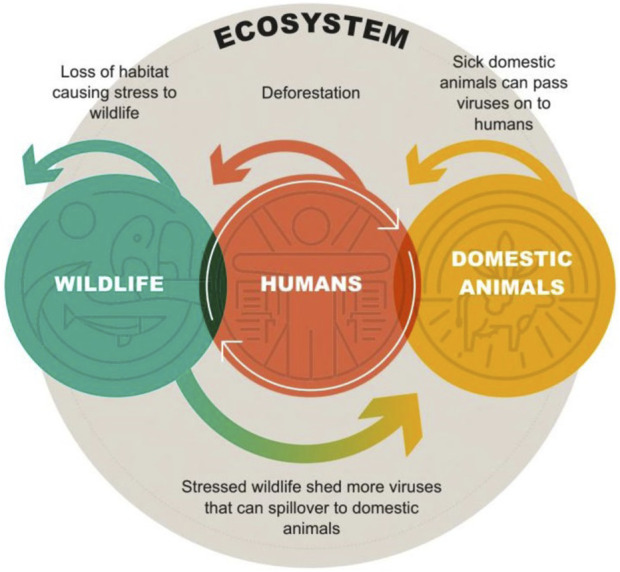
Conceptual example of the dynamic linkages between humans, wildlife, and domestic species within the ecosystem. In this example, deforestation caused by humans causes stress to wildlife from loss of habitat, which causes an increase in shed viruses that can spillover to domestic animals, who then pass viruses on to humans (from Ottinger and Geiselman, 2023).

The discussion and implementation of the One Health concept and framework has gained momentum over the last few decades. This framework has a historic foundation even in ancient religions and cultures, with the acknowledgement of the interactive relationship of man, nature, and health. More recently, the emphasis of One Health has been on the dynamics of pathogen transmission from wildlife to humans (for detailed discussion of history and literature, see Ottinger and Geiselman, 2023). As shown in Figure 8, the interrelationship of humans to their environment is multidimensional, involving impacts to the ecosystems as well as transfer of pathogens between humans, wildlife, and domestic species. This interactive co-dependence is best viewed conceptually as One Health or One Environmental Health for emphasis on wildlife. Further, the concept of One Environmental Health is highly relevant for assessing adverse outcomes for wildlife exposed to environmental metals and other contaminants as well as providing a critical linkage to implications for both wildlife and human health (Speer et al., 2019; Wise et al., 2019).

**FIGURE 9:**
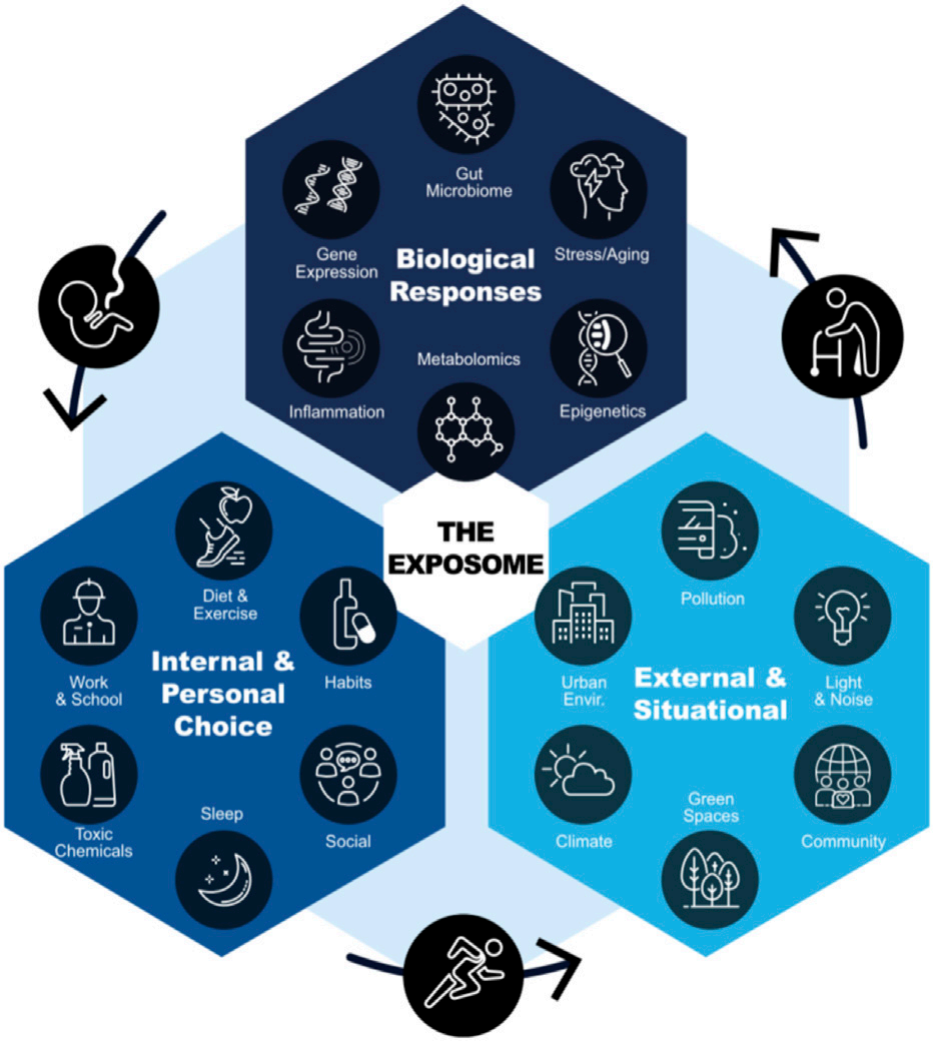
An individual’s Exposome is impacted by internal and personal choice, external and situational factors, and the biological responses to these factors throughout the lifespan (from Ottinger and Geiselman, 2023).

In contrast, the concept of the Exposome has gained increasing attention and provides an approach that considers human health as impacted by environmental factors (Sillé et al., 2020; Miller, 2023). The effects of lifelong exposures to environmental toxicants and other stressors build over the lifespan of an individual, resulting in an accumulation of deleterious environmental stressors (Dagnino, 2019; Niedzwiecki et al., 2019). This concept has broadened to include measures indicative of exposure and functional impacts, with consideration of differences in response with life stage (Nwanaju-Enwerem et al., 2021; Price et al., 2022). The ecosystem or wildlife health are not considered separately and often not as an influencing factor for a person’s Exposome. Originally defined to also include lifestyle factors (Wild, 2005), the Exposome is an exquisite cumulative measure for an individual that can serve as a potential predictive measure of both sides of the toxic aging coin. Exposomics describe a suite of molecular, epigenetic, endocrine, immunological, and other measures occurring in response to environmental exposures and stressors (Figure 9). Because each individual will vary in the extent of their response, it is important to have sufficient measures to establish that there is a problem (toxicants or environmental stressors) and then go on to develop risk assessments. These measures can be viewed chronologically as well as at selected points in time. As a result, the detection of a measurable adverse outcome may occur any time in an individual’s lifetime and this timing may vary with the individual.

The questions pertinent to the toxic aging coin are 1) Can we utilize a One Health or Exposome approach to identify/characterize causal drivers affecting aged populations and accelerating aging? and 2) Does incorporating an exposomics approach customized for wildlife provide the necessary data to identify and potentially predict adverse outcomes related to aging for wildlife?

There are ongoing activities listed below that address both questions, with the hope that there are sufficient data to obtain demographic information within and across species.

⇒ Long-term monitoring programs of known-age individuals to allow for detection of age-dependent effects,
⇒ Regional and local monitoring programs by scientists and citizen science groups, that document the individuals, species, productivity, and fledging, especially those that include band and resight protocols.
⇒ Environmental restoration in damaged areas (example: Gulf of Mexico Oil Spill in coastal areas) that document conditions and assess program success through evaluating health and longevity in known age individuals.
⇒ Validating molecular methods for sensitive measurement end points for exposures, which will provide an initial set of exposomics for wildlife.
⇒ Examine the data for exposures to traditional toxicants as well as to endocrine disrupting chemicals in both young, middle, and old populations, and
⇒ Synthesize datasets that have been collected regionally as well as in large geographic areas to provide the most complete information.

Both the One Health and Exposome concepts and their associated frameworks are relevant for the toxic aging coin perspective. In essence, both should place a greater emphasis on assessing wildlife as an integral part of ecosystem and human health. Additionally, the Exposomics used for human diagnostic and characterization of exposure should be customized for use in wildlife species, especially those with contaminated habitats and resource limitations. As human-wildlife conflict increases, the need for more information about the status of wildlife populations, ecosystems, and measures of exposure become more critical for management and for mitigation of adverse outcomes.

## 6 In summary: the toxic aging coin perspective and wildlife

The toxic aging coin provides a useful perspective for considering not only the effects of toxicants in a laboratory environment, but also the effects of a multitude of environmental stressors in the “field”. Given the rapid shift toward a large geriatric human population, this is a critical area of research for human health. The effects of environmental stressors on wildlife health across the lifespan are less understood, except for long-term monitoring of select populations. More long-term studies are critical for wildlife, with synthesis of data to understand the effects of environmental stressors on populations. Important data to collect include when aged individuals are still reproductively active and/or reproductive success is low (e.g., “slow” life history organisms), metadata on environmental conditions and climate effects. Information on human communities is important to assess the potential risk from human-wildlife conflict, including habitat loss, exposure to agrichemicals, and other stressors. It is important to have more studies on emerging contaminants and their effects on older people. Similarly, more studies on the effects of toxicological exposures are needed to understand risk and effects on across the lifespan in wildlife. We must incorporate the toxic aging coin perspective when considering the impacts of global challenges on humans and wildlife within a One Health framework.
